# TBI history and psychological symptom improvements following exercise therapies among U.S. service members

**DOI:** 10.3389/fnhum.2025.1638576

**Published:** 2025-08-29

**Authors:** Kristen H. Walter, Nicholas P. Otis, Laura D. Crocker, Hayley C. Myers, Kim T. Kobayashi Elliott, Betty Michalewicz-Kragh

**Affiliations:** ^1^Psychological Health and Readiness, Naval Health Research Center, San Diego, CA, United States; ^2^Leidos Inc., San Diego, CA, United States; ^3^Department of Public Health, Naval Medical Center San Diego, San Diego, CA, United States

**Keywords:** traumatic brain injury, depression, PTSD, physical activity, recreation therapy, exercise outside, nature exposure, military mental health

## Abstract

**Background:**

Major depressive disorder (MDD) is a prevalent and debilitating mental health disorder that is commonly comorbid with posttraumatic stress disorder (PTSD) and history of traumatic brain injury (TBI) in the U. S. military population. Exercise, particularly in natural environments, has been shown to effectively reduce depression and comorbid PTSD symptoms. However, little is known about whether history of TBI moderates symptom improvements following exercise interventions. Previous research has largely shown that military personnel with a history of TBI similarly benefited from evidence-based psychotherapy compared to those without a history of TBI. In contrast, no studies to date have compared those with and without a TBI history on symptom outcomes following exercise interventions.

**Methods:**

The present study is a secondary analysis of a clinical trial evaluating surf and hike therapies among active duty service members with MDD. Depression and PTSD symptom outcomes were compared between service members with (*n* = 47) and without (*n* = 48) a TBI history to determine whether TBI history moderated treatment response.

**Results:**

Multilevel modeling results indicated that history of TBI was not significantly related to change in depression symptoms over time (*p*s = 0.713–0.994). History of TBI was also not significantly associated with PTSD symptom severity from pre-to postprogram (*p* = 0.832); however, from preprogram through 3-month follow-up, service members without a history of TBI improved 14.7 points more than those with a TBI history (*p* = 0.018). Specifically, service members without a TBI history demonstrated continued improvement from postprogram to 3-month follow-up, while those with a TBI history maintained the gains achieved at postprogram. Potential explanatory factors, such as follow-up program attendance, physical activity levels, and concurrent mental health treatment, were investigated for their influence on this relationship and no significant effects emerged (*p*s = 0.143–0.822).

**Conclusion:**

Study findings showed that TBI history did not moderate depression outcomes, or PTSD outcomes from pre-to postprogram, following surf and hike therapies. However, service members without a history of TBI reported significantly greater improvements in PTSD symptom severity during the follow-up period, whereas those with a TBI history maintained their gains. Results suggest that while comparable in the short term, TBI history may reduce longer term PTSD symptom improvements following exercise interventions, such as surf and hike therapies.

## Introduction

Major depressive disorder (MDD) is a prevalent mental health disorder with serious negative consequences on individuals, families, and society. MDD is more prevalent among U.S. active duty military personnel and veterans relative to the general population, with meta-analytic estimates indicating rates as high as 23% in active duty service members and 20% in veterans (Moradi et al., 2021). Significant morbidity, disability, and health care utilization are associated with MDD (Bitter et al., 2024; Pappa et al., 2024; Yan et al., 2024), as well as reduced operational readiness and attrition from the military (Eliasson et al., 2012; Tankard et al., 2022). Furthermore, MDD is often comorbid with other conditions in military populations, particularly posttraumatic stress disorder (PTSD) and history of traumatic brain injury (TBI) (Hai et al., 2023; Kennedy et al., 2019). Active duty service members are at increased risk for sustaining a TBI relative to civilians (Reid and Velez, 2015), which can result from deployment (e.g., blasts from explosions) and non-deployment (e.g., contact sports, motor vehicle accidents, military training) mechanisms. In turn, TBI can increase the risk of developing MDD and PTSD, particularly in military populations (Loignon et al., 2020; Ponsford et al., 2018; Stein et al., 2015, 2019).

A TBI is defined as “a traumatically induced structural injury and/or physiological disruption of brain function as a result of an external force” (Veterans Affairs/Department of Defense Management and Rehabilitation of Post-Acute Mild Traumatic Brain Injury Work Group, 2021). TBIs are classified as mild, moderate, or severe, with approximately 82% being mild in severity (Traumatic Brain Injury Center of Excellence, 2024). The expected recovery of mild TBI (mTBI), or concussion, is a complete resolution of TBI-related cognitive, affective, and somatic sequelae and a return to baseline functioning within 3 months (Iverson, 2005; Porter and Johnson, 2020), though these post-concussive symptoms often resolve in less time (Karr et al., 2014). In fact, most service members who sustain an mTBI return to full duty within 10 to 14 days after rest and a progressive return to activity (Traumatic Brain Injury Center of Excellence, 2024). However, a portion continue to experience post-concussive symptoms beyond the acute phase (Mac Donald et al., 2017; Mac Donald et al., 2020; McDonald et al., 2021). Military personnel exhibit higher rates of prolonged recovery and persistent post-concussive symptoms compared to their civilian counterparts, which appears to be due in part to comorbid symptoms of MDD and PTSD (Belanger et al., 2010; Lange et al., 2014; Porter et al., 2018). Notably, post-concussive symptoms are non-specific and overlap with psychological symptoms, including those associated with MDD and PTSD (e.g., apathy, concentration difficulties, insomnia). Because of this clinical complexity, the VA/DoD clinical practice guideline for managing post-acute mTBI (2021) recommends that comorbid mental health conditions be evaluated and treated per their specific VA/DoD practice guidelines, regardless of mTBI history.

Consistent with clinical practice guidelines for the treatment of depression and PTSD, evidence-based psychotherapies (e.g., cognitive behavioral therapy) and pharmacological treatments (e.g., selective serotonin reuptake inhibitors) significantly reduce depression and PTSD symptoms (Forman-Hoffman et al., 2018; Simon et al., 2024) and are effective in individuals with history of TBI (Beedham et al., 2020; Cheng et al., 2021; Mikolić et al., 2019). However, there are notable limitations regarding treatment for MDD and PTSD in both military and civilian populations. Despite effective treatments, many individuals drop out of treatment prematurely, do not adequately respond to treatment, or relapse after treatment completion (Semmlinger et al., 2024; Varker et al., 2021; Wojnarowski et al., 2019). Notably, military personnel appear to have reduced response and higher rates of dropout relative to civilians (Kitchiner et al., 2019; Varker et al., 2021). Furthermore, a significant portion of military personnel with depression and other mental health disorders do not access mental health services (Thériault et al., 2020). Only one third of service members with depression or PTSD symptoms reported any mental healthcare utilization in the previous year (Sharifian et al., 2025). There are often notable barriers to accessing evidence-based care, including attitudinal barriers, such as stigma, and structural barriers, such as cost and time demands (Andrade et al., 2014; Hom et al., 2017), which may be further compounded for active duty service members. For example, over one third of service members reported that seeking mental health services damages one’s military career (Meadows et al., 2021). Therefore, a critical need exists for other stand alone or adjunctive interventions for mental health conditions to address barriers to care and improve treatment outcomes.

Exercise is one such intervention shown to be an effective primary and adjunctive treatment for depression and PTSD symptoms across various populations (Björkman and Ekblom, 2022; Ding et al., 2025; Hegberg et al., 2019; Moran et al., 2024; Morres et al., 2018; Noetel et al., 2024; Otis et al., 2024; Walter et al., 2023a, 2023b). Exercise interventions may also be well suited for the military population as physical fitness is a requirement for duty, and service members have reported high satisfaction with such interventions (Walter et al., 2023a). When delivering exercise interventions, certain contextual factors have been shown to enhance outcomes. For example, exercise in natural, outdoor settings appears to offer superior mental health benefits compared with exercise indoors or in outdoor urban settings (Chen et al., 2025; Thompson Coon et al., 2011; Wicks et al., 2022). Exposure to natural environments while exercising enhances an array of psychological outcomes, including depression, anxiety, negative affect, anger, energy, and positive affect (Ma et al., 2024; Noseworthy et al., 2023; Thompson Coon et al., 2011; Wicks et al., 2022). Another contextual factor is group-based exercise, which provides opportunities for social interaction and connection (Britton et al., 2020; Mason and Holt, 2012). In a randomized clinical trial that combined these factors by comparing group-based exercise interventions in the natural environment (i.e., surf and hike therapies) for U. S. service members with MDD, results revealed significant improvements in depression, PTSD, anxiety, negative affect, psychological resilience, and social functioning that were comparable between exercise interventions (Walter et al., 2023a, 2023b; Otis et al., 2024). Similar reductions in depression symptoms were demonstrated following a surf therapy program for Australian service members with MDD (Moran et al., 2024). Collectively, exercise interventions can effectively reduce psychological symptoms and may provide a desirable treatment approach for certain populations, including military personnel.

However, it is unclear whether a history of TBI moderates symptom improvements from exercise interventions among military populations. Studies have shown that exercise is feasible and beneficial for reducing depression in individuals with a history of TBI (Liu et al., 2019; Perry et al., 2020), but no studies to date have directly compared those with and without a history of TBI on mental health outcomes following exercise interventions. In contrast to the limited understanding of exercise interventions, psychotherapy research shows comparable benefits for service members and veterans with and without history of TBI following evidence-based treatments (Bomyea et al., 2017; Ragsdale and Voss Horrell, 2016; Sripada et al., 2013; Wachen et al., 2022), with symptom reductions in those with a history of TBI mirroring those observed in the broader PTSD treatment literature (e.g., Chard et al., 2011; Jak et al., 2019; Walter et al., 2014; Wolf et al., 2015). Although outcomes following treatment generally demonstrate similar symptom reductions between military personnel with and without a TBI history, data on treatment attendance are mixed. Specifically, research findings demonstrated no significant differences between TBI groups on session attendance (Bomyea et al., 2017; Sripada et al., 2013), trends for those with a TBI history to attend fewer sessions (Davis et al., 2013), and significant effects of TBI history in predicting greater dropout and lower session attendance (Berke et al., 2019). Taken together, findings suggest comparable symptom reductions following psychotherapy among military personnel with and without a TBI history, but that there may be lower engagement among those with a TBI history.

Considering limitations in access and response to evidence-based psychotherapies, coupled with the high prevalence of TBIs among military personnel, it is critical to understand whether TBI history influences psychological outcomes of other treatments for MDD, such as exercise. To date, no studies have directly compared those with and without TBI history on psychological outcomes following exercise interventions. Therefore, this study aimed to address this significant gap in the literature by conducting a secondary analysis of a clinical trial evaluating surf and hike therapies among active duty service members with MDD. Specifically, we compared depression and PTSD symptom outcomes between those with and without a history of TBI to determine whether TBI history moderated exercise treatment response. Hypotheses were exploratory given the lack of comparative effectiveness data regarding TBI history in the exercise intervention literature to base a prediction. Understanding whether TBI history affects exercise intervention response can inform care decisions and identify the most effective treatment approaches for service members with MDD to decrease their symptoms and improve operational readiness.

## Methods

### Participants

The parent study consisted of 96 U.S. active duty service members referred to the Wounded, Ill, and Injured (WII) Wellness Program at Naval Medical Center San Diego (NMCSD). Ninety-five service members with complete TBI data were included in this study. Service members were eligible for study participation if they met diagnostic MDD criteria per the clinician-administered Mini International Neuropsychiatric Interview (MINI) (Sheehan et al., 1998) and had not previously participated in the NMCSD Surf or Hike Therapy programs. All service members provided voluntary, written informed consent and received clearance to participate from medical providers at NMCSD.

### Program

The Surf and Hike Therapy programs were delivered as a standard care option at NMCSD. Each program offered six weekly sessions lasting 3–4 h in duration. The programs used a cohort format, with approximately 20 service members per cycle. A public beach in San Diego served as the location for Surf Therapy, and various locations throughout San Diego County were used for Hike Therapy. For both programs, the activity itself was considered the therapeutic element (e.g., Hawkins et al., 2016) and there was no separate psychotherapeutic component.

### Procedure

Within 2 weeks prior to the program, service members completed a preprogram assessment that consisted of clinical interview and self-report measures. Once eligibility was determined, service members were randomly assigned to either Surf or Hike Therapy. Service members completed a postprogram assessment within 2 weeks of completing the program, and a follow-up assessment at 3 months. Brief self-report assessments were also completed before and after each exercise session. The study assessor was blinded to service members’ intervention condition. Service members were not financially compensated for study participation but were allowed to keep the Fitbit device used for secondary data collection if the device was worn for at least 50% of the study period. Study procedures were approved by the NMCSD Institutional Review Board and performed in compliance with all applicable federal regulations governing the protection of human subjects. For additional study details, please (see Walter et al., 2019, 2023a).

### Measures

The preprogram assessment included clinical interview and self-report measures of participant demographics, service characteristics, concurrent psychotherapy and/or pharmacotherapy for depression, depression and PTSD symptom severity, physical activity, and TBI history. Postprogram and 3-month follow-up assessments included clinical interview measures and self-reported concurrent treatments and symptom severity. Exercise session assessments measured self-reported depression and anxiety symptom severity.

The Montgomery Åsberg Depression Rating Scale (MADRS) (Montgomery and Åsberg, 1979) is a clinician-administered measure of depression symptom severity. The MADRS contains 10 items rated from 0 to 6 and then summed, with higher total scores indicating greater symptom severity. The MADRS was administered at preprogram, postprogram, and the 3-month follow-up. Internal consistency for the MADRS was high across these assessment time points (*α* = 0.74 to 0.90).

The nine-item Patient Health Questionnaire (PHQ-9) (Kroenke et al., 2001) assessed self-reported depression symptom severity. Each item is rated from 0 to 3 and subsequently summed; higher total scores signify greater symptom severity. The PHQ-9 was completed at preprogram, postprogram, and 3-month follow-up. Internal consistency was high across assessment time points for the PHQ-9 (α = 0.77 to 0.89).

The PTSD Checklist for DSM-5 (PCL-5) (Weathers et al., 2013a) with extended Life Events Checklist (Weathers et al., 2013b) evaluated PTSD symptom severity and self-reported trauma history, respectively. The measure contains 20 questions rated on a 0 to 4 scale and summed, with higher total scores indicating greater PTSD symptom severity. The PCL-5 was completed at preprogram, postprogram, and 3-month follow-up. High internal consistency was demonstrated for the PCL-5 across assessment time points (α = 0.87 to 0.97).

The four-item Patient Health Questionnaire (PHQ-4) (Kroenke et al., 2009) measured self-reported depression and anxiety symptom severity. Each item is rated on a scale from 0 to 3 and then summed, with higher total scores reflecting greater depression and anxiety symptom severity. The PHQ-4 was completed before and after each exercise session. Internal consistency for the PHQ-4 ranged from α = 0.77 to α = 0.89.

The Ohio State TBI Identification Method–Short Form (OSU TBI-ID-SF) (Corrigan and Bogner, 2007) is a gold standard, structured interview used to evaluate lifetime history of TBI and was administered at the pretreatment assessment. Interview queries included injuries to the head or neck that resulted in hospitalization or emergency room treatment, and injuries as a result of vehicular accidents, falls, being struck by objects, sports, fights, or exposure to explosions. For each reported injury, participants report loss of consciousness (LOC), memory gaps, or confusion at the time of the injury (i.e., posttraumatic amnesia or alteration of consciousness), and the duration of any LOC. Summary indices are calculated to reflect the likelihood of lifetime TBI, as well as TBI severity, number of TBIs, age at first TBI with LOC, and occurrence of multiple TBIs. Definitions of TBI severity are based on Veterans Affairs/Department of Defense Management and Rehabilitation of Post-Acute Mild Traumatic Brain Injury Work Group (2021) recommendations and include mild, moderate, and severe. In this study, TBI groups were combined because subsamples of mild (*n* = 40) and moderate (*n* = 7) TBI (based on worst injury) were limited in number and mild to moderate TBIs are often examined together in treatment research (e.g., Bomyea et al., 2017; Jak et al., 2019; Wolf et al., 2012). Therefore, TBI history was dichotomized into TBI (*n* = 47) and no TBI (*n* = 48) for analysis. There were no participants in this study who endorsed a history of severe TBI.

### Data analysis

Descriptive statistics established sample and preprogram characteristics. To examine descriptive differences between TBI groups, *t*-tests were used for continuous variables and chi-square tests for categorical variables. Multilevel modeling (MLM) determined whether TBI history moderated depression and PTSD symptom outcomes. MLMs used a step-up model-building process; logical covariance matrices were compared and selected based on model fit according to the Akaike information criterion with respect to the number of parameters specified. All final models used restricted maximum likelihood to account for missing data.

For models measuring symptom change from preprogram through 3-month follow-up, time was set as a repeated effect with an unstructured covariance matrix. Both time (2 = preprogram, 1 = postprogram, and 0 = 3-month follow-up) and TBI history (1 = no TBI, 0 = TBI) were used as fixed effects, along with their interaction term. Separate models were run for outcomes of the MADRS (*n* = 94), PHQ-9 (*n* = 95), and PCL-5 (participants with a PTSD diagnosis only, *n* = 51).

For models measuring symptom change within exercise sessions, time was set as a random effect by subject with a first-order autoregressive covariance matrix, along with the intercept, week of session, and a crossed effect of time × week of session. Time × week of session was also set as a repeated effect of subject and used a compound symmetry covariance matrix. Time (1 = postsession, 0 = presession), week of session (1–6), and TBI history (1 = no TBI, 0 = TBI) were used as fixed effects, along with the interaction terms of time × TBI history and time × week of session. The final model for the PHQ-4 included 86 participants.

Due to power concerns, analyses collapsed across exercise interventions (i.e., surf and hike therapies) and precluded exploration of differential treatment response by TBI history. Similar symptom and functional improvements were observed between surf and hike therapies in prior work (Walter et al., 2023a, 2023b). SPSS Version 29 (IBM, Armonk, NY) was used for all analyses.

## Results

Table 1 displays sample characteristics overall and by TBI group. Most service members were White (42.1%), female (52.6%), senior enlisted (i.e., ranks E-5 to E-9; 59.4%), and received concurrent pharmacotherapy for depression (65.3%). Participants were also relatively young (*M* = 28.2 years, *SD* = 5.6), which is representative of the U.S. active duty military population (DoD, 2024). Forty-nine percent (*n* = 47) of service members endorsed a history of TBI, and among those, 85.1% (*n* = 40) were of mild severity, 14.9% (*n* = 7) moderate severity, and none were severe. The mean duration of time since the most recent TBI was 7.5 years (*SD* = 0.4) and ranged from 1 to 21 years. Most service members with a TBI history reported multiple TBIs (*M* = 2.96, *SD* = 1.83; range 1–9), with 87.2% (*n* = 41) endorsing two or more TBIs. At preprogram, the overall sample reported *moderate* depression symptom severity on the MADRS (*M* = 27.0, *SD* = 8.4) and *moderately severe* symptom severity on the PHQ-9 (*M* = 17.1, *SD* = 4.9). Those with PTSD (*n* = 57) reported an average PCL-5 score of 50.3 (*SD* = 13.3), exceeding the suggested clinical cutoff of 33 as shown among treatment-seeking service members (Wortmann et al., 2016). Forty percent (*n* = 38) of the sample reported comorbid diagnoses of MDD and PTSD with a TBI history. Most demographic and preprogram variables did not significantly differ based on TBI group (*p*s > 0.202). However, there was an exception: a positive TBI history was significantly related to PTSD diagnostic status at preprogram (*p* < 0.001). Of note, positive TBI history was also significantly related to PTSD diagnostic status at postprogram (*p* = 0.046) and 3-month follow-up (*p* = 0.002). The TBI groups also did not significantly differ on compliance and engagement variables, such as the number of exercise therapy sessions attended, program completion rates, and completion of postprogram and 3-month assessments (*p*s ≥ 0.237).

**Table 1:** Sample characteristics and traumatic brain injury (TBI) group comparisons.

Characteristic	Total Sample	TBI	No TBI	*p*
	*N* = 95	*n* = 47	*n* = 48	
Age, years, *M* (*SD*)	28.2 (5.6)	28.8 (5.5)	27.6 (5.7)	0.291
Sex, *n* (%)				0.756
Female	49 (51.6)	25 (53.2)	24 (50.0)	
Male	46 (48.4)	22 (46.8)	24 (50.0)	
Race/ethnicity, *n* (%)				--
Asian or Asian American	3 (3.2)	1 (2.1)	2 (4.2)	
Black or African American	15 (15.8)	8 (17.0)	7 (14.6)	
Hispanic, Latino, or Spanish origin	17 (17.9)	5 (10.6)	12 (25.0)	
Multiracial	19 (20.0)	12 (25.5)	7 (14.6)	
Native American or Alaska Native	1 (1.1)	0 (0.0)	1 (2.1)	
White	40 (42.1)	21 (44.7)	19 (39.6)	
Rank, *n* (%)				--
E1–E4	33 (34.7)	16 (34.0)	17 (35.4)	
E5–E9	57 (60.0)	29 (61.7)	28 (58.4)	
Officer	5 (5.3)	2 (4.3)	3 (6.3)	
Randomized exercise therapy condition, *n* (%)				0.917
Surf Therapy	48 (50.5)	24 (51.5)	24 (50.0)	
Hike Therapy	47 (49.5)	23 (48.9)	24 (50.0)	
Sessions attended, *M* (*SD*)^a,b^	3.9 (1.6)	4.1 (1.3)	3.8 (1.6)	0.374
Completion of assigned program, *n* (%)^b,c^	67 (77.0)	31 (75.6)	36 (78.3)	0.765
Completion of postprogram assessment, *n (%)*	87 (91.6)	42 (89.4)	45 (93.8)	0.486
Completion of 3-month assessment, *n (%)*	74 (77.9)	39 (83.0)	35 (72.9)	0.237
Activity level, *n* (%)^d^				--
Low/inactive (<600 MET mins/wk)	10 (10.5)	7 (14.9)	3 (6.3)	
Moderately active (600–2,999 MET mins/wk)	37 (38.9)	19 (40.4)	18 (37.5)	
Highly active (≥3,000 MET mins/wk)	31 (32.6)	12 (25.5)	19 (39.6)	
Concurrent pharmacotherapy for depression, *n* (%)	62 (65.3)	28 (59.6)	34 (70.8)	0.249
PTSD diagnosis, *n* (%)	57 (60.0)	38 (80.9)	19 (39.6)	<0.001
Symptom severity, preprogram, *M* (*SD*)				
MADRS	26.9 (8.5)	28.1 (8.4)	25.8 (8.5)	0.202
PHQ-9	17.1 (4.9)	17.7 (4.5)	16.5 (5.3)	0.227
PCL-5^e^	50.5 (13.3)	50.4 (12.7)	50.9 (14.6)	0.889
Worst TBI severity, *n* (%)				--
Mild	--	40 (85.1)	--	
Moderate	--	7 (14.9)	--	
Severe	--	0 (0.0)	--	
Years since most recent TBI, *M* (SD)	--	7.5 (0.4)	--	--
Number of TBIs, *M* (SD)	--	3.0 (1.8)	--	--

E, enlisted rank; MET mins, metabolic equivalent of task minutes; PTSD, posttraumatic stress disorder; MADRS, Montgomery–Åsberg Depression Ranking Scale; PHQ-9, 9-item Patient Health Questionnaire; PCL-5, PTSD Checklist for DSM-5. Counts vary based on missing data; percentages are based on full samples for that column.

^a^For fidelity, only sessions in which the assigned modality was conducted are included. Occasionally, due to adverse weather, sessions consisted of alternative activities (e.g., visit to the National Surf Museum in Surf Therapy, strength and onditioning class in Hiking Therapy).

^b^Because programs were halted due to the sudden onset of COVID-19, participants (*n* = 8) in the affected cohort were not counted in completion or attendance statistics.

^c^Program completion was defined by the Naval Medical Center San Diego as missing no more than two sessions of the assigned modality.

^d^Preprogram physical activity level data were calculated (see IPAQ Research Committee, 2005) from the self-report version of the International Physical Activity Questionnaire–Short Form.

^e^Reported only for those with a diagnosis of PTSD.

**Figure 1:**
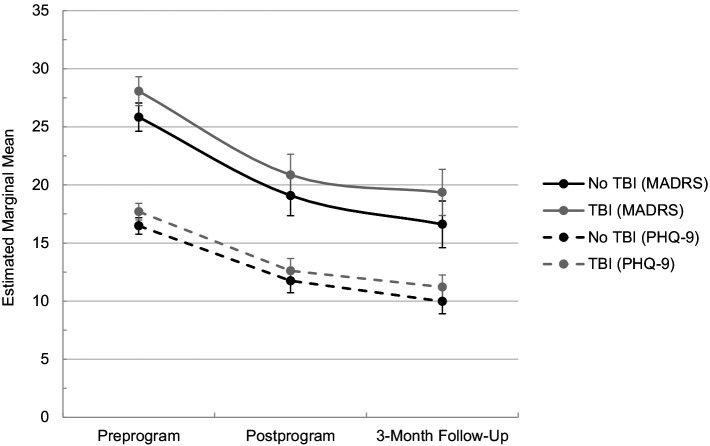
Depression estimated marginal means over time. *N* = 95; TBI, traumatic brain injury; MADRS, Montgomery-Åsberg Depression Rating Scale; PHQ-9, 9-item Patient Health Questionnaire.

Tables 2–4 display estimated marginal means from MLMs for each outcome measure from preprogram through 3-month follow-up. In addition, the tables display within-and between-TBI-group differences in these scores. MLM results revealed that TBI history did not significantly moderate changes in depression symptoms over time. Specifically, no significant differences in depression symptom change were observed between TBI groups on the MADRS (pre to post: *p* = 0.844; pre to follow-up: *p* = 0.855; Table 2) or PHQ-9 (pre to post: *p* = 0.781; pre to follow-up: *p* = 0.994; Table 3). For a graphical depiction of these changes in depression scores, see Figure 1. Session analyses similarly revealed that TBI history did not influence PHQ-4 scores from pre-to postsession (*p* = 0.713; see Table 5).

**Table 2:** MADRS estimated marginal means (EMMs) within and between traumatic brain injury (TBI) groups.

Group and time period	Time point	EMM	Within-group difference (95% CI)	*p*	Between-group difference (95% CI)	*p*
Pre-post						
No TBI	Pre	25.83	−6.75 (−10.42, −3.08)	<0.001	−0.46 (−5.05, 4.14)	0.844
	Post	19.09				
TBI	Pre	28.07	−7.21 (−11.00, −3.41)	<0.001		
	Post	20.86				
Pre-3 mo						
No TBI	Pre	25.83	−9.22 (−13.74, −4.70)	<0.001	0.51 (−5.02, 6.05)	0.855
	3 mo	16.61				
TBI	Pre	28.07	−8.71 (−13.18, −4.24)	<0.001		
	3 mo	19.36				

*N* = 95; MADRS, Montgomery–Åsberg Depression Ranking Scale; CI, confidence interval.

**Table 3:** PHQ-9 estimated marginal means (EMMs) within and between traumatic brain injury (TBI) groups.

Group and time period	Time point	EMM	Within-group difference (95% CI)	*p*	Between-group difference (95% CI)	*p*
Pre-post						
No TBI	Pre	16.48	−4.72 (−6.86, −2.58)	<0.001	−0.38 (−3.05, 2.30)	0.781
	Post	11.76				
TBI	Pre	17.70	−5.10 (−7.31, −2.89)	<0.001		
	Post	12.61				
Pre-3 mo						
No TBI	Pre	16.48	−6.50 (−8.95, −4.05)	<0.001	0.01 (−2.98, 3.01)	0.994
	3 mo	9.98				
TBI	Pre	17.70	−6.49 (−8.90, −4.08)	<0.001		
	3 mo	11.21				

*N* = 95; PHQ-9, 9-item Patient Health Questionnaire; CI, confidence interval.

**Table 4:** PCL-5 estimated marginal means (EMMs) within and between traumatic brain injury (TBI) groups over time.

Group and time period	Time point	EMM	Within-group difference (95% CI)	*p*	Between-group difference (95% CI)	*p*
Pre-post						
No TBI	Pre	50.90	−17.60 (−26.76, −8.44)	<0.001	1.02 (−8.64, 10.69)	0.832
	Post	33.26				
TBI	Pre	50.37	−16.58 (−22.87, −10.28)	<0.001		
	Post	33.79				
Pre-3 mo						
No TBI	Pre	50.90	−30.79 (−42.48, −19.10)	<0.001	14.71 (2.66, 26.76)	0.018
	3 mo	20.11				
TBI	Pre	50.37	−16.08 (−23.55, −8.61)	<0.001		
	3 mo	34.29				

*n* = 57; PCL-5, PTSD Checklist for DSM-5; CI, confidence interval.

**Table 5:** PHQ-4 estimated marginal means (EMMs) within and between traumatic brain injury (TBI) groups within session.

Group	Time point	EMM	Within-group difference (95% CI)	*p*	Between-group difference (95% CI)	*p*
No TBI	Presession	5.60	−2.88 (−3.32, −2.45)	<0.001	−0.12 (−0.73, 0.50)	0.713
	Postsession	2.71				
TBI	Presession	6.20	−3.00 (−3.46, −2.55)	<0.001		
	Postsession	3.20				

*N* = 95; PHQ-4, 4-item Patient Health Questionnaire; CI, confidence interval.

**Figure 2:**
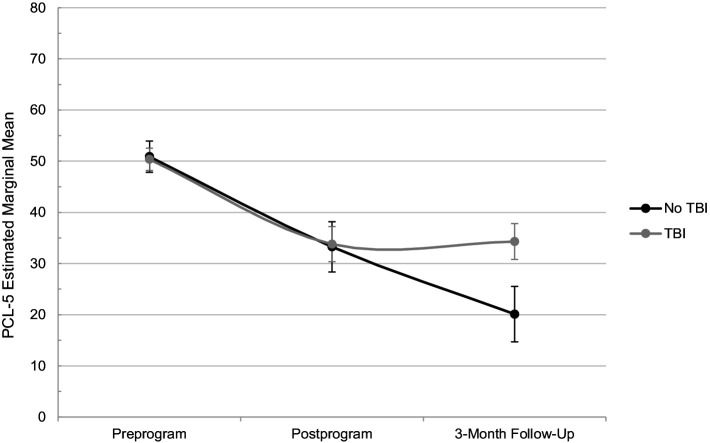
PTSD estimated marginal means over time. *n* = 57; TBI, traumatic brain injury; PCL-5, PTSD Checklist for DSM-5.

For PTSD symptom change, TBI history did not significantly influence PCL-5 scores from pre-to postprogram (*p* = 0.832; Table 4); however, a significant moderation effect was observed over the longer term. From preprogram to 3-month follow-up, service members without a TBI history improved 14.7 points more than those with a TBI history (*p* = 0.018). Specifically, service members without a TBI history significantly decreased PCL-5 scores from 50.9 at preprogram to 20.1 at the 3-month follow-up (*MD* = 30.8, *p* < 0.001); those with a TBI significantly decreased from 50.4 to 34.3 over the same period (*MD* = 16.1, *p* < 0.001). Figure 2 depicts this difference graphically over time, showing that service members without a TBI history demonstrated continued improvement from postprogram to 3-month follow-up, while those with a TBI history maintained the gains achieved at postprogram. Potential explanatory factors, such as follow-up program attendance, physical activity levels, and concurrent pharmacotherapy treatment, were investigated for their influence on this relationship. However, no significant effects emerged (*p*s = 0.143–0.822).

Additionally, because the TBI group consisted of both mild (*n* = 40) and moderate (*n* = 7) TBI severity subgroups, sensitivity analyses were run for all outcomes (MADRS, PHQ-9, PHQ-4, PCL-5) that excluded participants with moderate TBI. These sensitivity analyses aimed to determine whether those with a moderate TBI accounted for observed results; outcome patterns were analogous to original findings.

## Discussion

History of TBI is common among service members, frequently comorbid with psychiatric diagnoses such as PTSD and MDD, and a potential moderator of treatment outcomes. In this sample of service members with MDD, approximately half reported a TBI history; these were predominantly mild (85%), consistent with data from Traumatic Brain Injury Center of Excellence (2024). Among those who reported a TBI history, service members primarily sustained their most recent TBI over 1 year ago (91%), after most recovery would be expected to have occurred (Iverson, 2005; Karr et al., 2014; Porter and Johnson, 2020). However, most service members with a reported TBI history endorsed multiple TBIs, which could result in more severe symptoms and impairments (Reid and Velez, 2015; Vanderploeg et al., 2012). By most study metrics, service members with MDD with and without a history of TBI were similar in this sample. One notable exception was a strong relationship between TBI history and PTSD diagnosis, which was found at each time point and aligned with prior work (Davis et al., 2013; Loignon et al., 2020; Ponsford et al., 2018; Stein et al., 2015, 2019). Further, 40% of the current sample reported comorbid diagnoses of MDD and PTSD with a TBI history, which are commonly co-occurring conditions with overlapping symptoms.

Due to the difficulty determining the etiology of symptoms for those with psychological symptoms and a TBI history, it is recommended that the focus of clinical care be on alleviating presenting symptoms (Veterans Affairs/Department of Defense Management and Rehabilitation of Post-Acute Mild Traumatic Brain Injury Work Group, 2021) with treatments such as evidence-based psychotherapies. In addition, exercise interventions significantly improve depression and PTSD symptoms (Björkman and Ekblom, 2022; Hegberg et al., 2019; Moran et al., 2024; Morres et al., 2018; Noetel et al., 2024; Otis et al., 2024; Walter et al., 2023a, 2023b) and may offer a suitable treatment option for service members given the emphasis on physical fitness, which is a requirement for active duty service. The present study advances understanding of exercise interventions by evaluating whether a history of TBI moderated symptom outcomes. Study results demonstrated that service members with MDD, with and without a history of TBI, showed comparable reductions in depression symptom severity over time, across both treatment and follow-up periods. These findings are encouraging and suggest that regardless of TBI history, service members with MDD can clinically improve their depression severity following surf and hike therapies. The lack of moderation by TBI history could reflect the effectiveness of exercise on depression symptoms (Morres et al., 2018; Schuch et al., 2016). More specifically, exercise reduces depression through physiological mechanisms (e.g., neuroplasticity, inflammation) and psychosocial mechanism, such as improvements in self-efficacy, self-esteem, social support, and connection (Kandola et al., 2019). Furthermore, exercise interventions may serve as behavioral activation, an evidence-based treatment approach for depression designed to increase engagement in enjoyable, meaningful, and reinforcing activities (Cuijpers et al., 2007; Lewinsohn et al., 1967). Surf and hike therapies may effectively rely on this approach in conjunction with the physiological and psychosocial benefits to reduce depression symptoms over time, irrespective of TBI history and symptom etiology.

For service members with MDD and comorbid PTSD symptoms, pre- to posttreatment findings mirrored those for depression, where similar trajectories of symptom improvement were observed for those with and without a TBI history. The results from the treatment period align with the psychotherapy literature in that TBI history does not attenuate treatment response (Bomyea et al., 2017; Crocker et al., 2019; Ragsdale and Voss Horrell, 2016; Sripada et al., 2013; Wachen et al., 2022). However, findings diverged in the 3 months following the program, where service members without a TBI history showed further improvement in PTSD symptoms while those with a TBI history demonstrated maintenance of gains (but not additional symptom improvement). Additional exploration of this relationship did not reveal significant effects based on whether service members attended exercise sessions in the follow-up period, physical activity levels, or concurrent mental health treatment. However, it should be noted that service members with a TBI history showed higher rates of comorbid PTSD diagnoses at all three assessment time points. Results indicate that perhaps a TBI history interferes with continued PTSD symptom improvement following exercise interventions or may have been accounted for by factors beyond the scope of this study. It also may be the case that for those with a TBI history and comorbid PTSD, the exercise interventions largely improved non-specific symptoms of PTSD that overlap with MDD (e.g., negative affect, anhedonia, feeling detached from others) but did not specifically address and notably improve symptoms that are unique to PTSD and/or more trauma related (e.g., intrusions, avoidance of trauma-related stimuli, trauma-related guilt and cognitions). Clinical implications of these findings suggest that exercise interventions can significantly reduce PTSD symptoms; however, to achieve further reductions in PTSD symptoms following these interventions, service members with a history of TBI and comorbid PTSD may benefit from trauma-focused treatment (e.g., Norman et al., 2021; Walter et al., 2014).

Study results should be taken with consideration of study limitations. TBI history was determined by use of an established interview measure rather than medical records from the time of injury. However, interview measures are a methodologically sound approach to TBI assessment, given that medical records from the time of injury are seldom available and are advantageous compared with other options, such as self-report instruments. While participants reported the most common levels of TBI severity (i.e., mild and moderate), study results likely do not generalize to those with a severe TBI history and greater impairments. Due to power concerns, analyses collapsed across exercise interventions (i.e., surf and hike therapies) and precluded exploration of differential treatment response by TBI history. Similar symptom and functional improvements were observed between surf and hike therapies in prior work (Walter et al., 2023a, 2023b); however, it is possible that with greater power, one intervention could yield greater benefits based on TBI history compared with the other. The sample was composed of service members who were at least moderately physically active (72%) and relatively young (average age of 28 years) at the preprogram assessment, so findings may not extend to the general U. S. population with depression who tends to be less physically active and older. Most service members received concurrent treatment (e.g., psychiatric medications, psychotherapy) during the surf and hike therapy programs, and it is not possible to fully disentangle the unique effects of treatments received. Lastly, symptom change within individual exercise sessions was only assessed for depression and not PTSD due to the focus of the parent study on MDD.

The study also provides several strengths. Multimethod assessment measures that were used included gold-standard interview (i.e., MADRS; MINI; OSU-TBI) and/or validated self-report measures (i.e., PHQ-9; PHQ-4; PCL-5) to determine psychological diagnoses, symptoms, and TBI history. Furthermore, longitudinal assessments were repeatedly administered from pretreatment through the 3-month follow-up allowing for symptom changes to be examined over time. Study methodology featured a pragmatic trial design (Beidas et al., 2023) that applied a rigorous, randomized design in the naturalistic Military Health System setting where the Surf and Hike Therapy programs were offered. To account for factors that could affect symptom outcomes following the exercise interventions, concurrent mental health treatments, additional exercise sessions in the follow-up period, and physical activity levels were assessed and empirically examined. Finally, this was the first study, to our knowledge, to explore whether TBI history moderated psychological symptoms following exercise interventions, which extends work conducted in both the psychotherapy and exercise literatures.

In sum, study findings showed that a TBI history did not moderate depression or PTSD symptoms from pre-to posttreatment among service members with MDD who received either Surf or Hike Therapy. Results diverged from posttreatment to 3-month follow-up, in that for depression symptoms, service members with and without a TBI history both demonstrated continued symptom improvements. Whereas for PTSD symptoms, service members with a TBI history maintained their symptom improvements while those without such history showed further improvement. An encouraging finding, and one consistent with similar research conducted with evidence-based psychotherapy, is that TBI history did not appear to moderate overall symptom change during treatment or the maintenance of gains following exercise-based interventions—both groups significantly improved or maintained gains. The one advantage for service members without a TBI history involved PTSD symptom reduction in the follow-up period, where they reported continued benefit compared to those with a TBI history. Further research is needed to both replicate and further disentangle this finding by determining whether it is a direct or indirect effect of TBI history. Additionally, exploring whether evidence-based, trauma-focused treatments could further reduce PTSD symptoms following exercise-based interventions would inform precision medicine for service members with MDD, PTSD, and a history of TBI.

## Data Availability

The datasets generated and/or analyzed during the current study are not publicly available due to personally identifiable information regulations, but they may be made available by the corresponding author on reasonable request and approval by the Naval Medical Center San Diego Institutional Review Board/Privacy Office.

## Author disclaimer

KW, KK, and BM-K are employees of the U.S. Government. This work was prepared as part of their official duties. Title 17, U.S.C. §105 provides that copyright protection under this title is not available for any work of the U.S. Government. Title 17, U.S.C. §101 defines a U.S. Government work as work prepared by a military service member or employee of the U.S. Government as part of that person’s official duties. Report No. 25–43 was supported by the U.S. Navy Bureau of Medicine and Surgery under work unit no. N1600. The views expressed in this article are those of the authors and do not necessarily reflect the official policy or position of the Department of the Navy, Department of Defense, nor the U.S. Government. The study protocol was approved by the Naval Medical Center San Diego Institutional Review Board in compliance with all applicable federal regulations governing the protection of human subjects. Research data were derived from approved Naval Medical Center San Diego Institutional Review Board protocol number NMCSD.2017.0007.
